# Why the Watchdog Won’t Bite: U.S. Food and Drug Administration Challenges

**DOI:** 10.5811/westjem.2016.9.32492

**Published:** 2016-11-04

**Authors:** Joe Lex

**Affiliations:** Lewis Katz School of Medicine at Temple University, Department of Clinical Emergency Medicine, Philadelphia, Pennsylvania

For many years before I retired, I gave a talk and wrote an essay about New Drugs and Devices.^1^ Each year I learned more about interpreting the medical literature and more about the United States Food and Drug Administration (FDA).

I was curious about why so many drugs were approved, only to be found useless – or worse, harmful – in practice, and even pulled from the market. I was fascinated by drugs that were approved despite offering no advantages over what was already available. I was mystified about a system in which the fox seemed to be guarding the henhouse. I read books by former editors^2^ in which they admitted that the pharmaceutical industry controlled medical journal content in innumerable and unimaginable ways.

It took a disaster to bring the FDA into existence. The so-called “Massengill massacre” in 1937 caused deaths in more than 100 patients who took “elixir sulfanilamide,” which had used the poisonous diethylene glycol as an excipient. This stimulated the U.S. government to give the FDA power to oversee data for the approval of drugs, as well as many foods, medical devices, and cosmetics.^3^ Its finest hour may have come in 1962 when a stubborn physician/pharmacologist, Frances Oldham Kelsey, insisted on seeing better safety data for a drug used widely in Europe to treat “morning sickness” before she would allow it to be approved in the U.S. When the tragedy of thalidomide became known, the FDA was lauded for its cautious reasoning.^4^

During the 1980s’ AIDS epidemic, the FDA was vilified by activists who initially misunderstood its function.^5^ It is not the job of the FDA to develop and test new drugs, or to set their prices, but to evaluate the data presented to it by drug manufacturers and cautiously recommend approval or no approval. The Prescription Drug User Funding Act (PDUFA) of 1992 started shifting the manner in which the FDA was funded. Requiring commercial entities to pay for their own oversight agency sounds like an odd way to do business, but it is not uncommon in the U.S. where agencies such as Customs and Immigration Services (CIS) and the Federal Communication Commission (FCC) also pay for their own oversight.^6^

The FDA is an imperfect watchdog. Its bite is not quite toothless, but it does little damage. Eli Lilly’s olanzapine (Zyprexa®) has been implicated in hundreds of deaths and thousands of cases of metabolic syndrome; fines were paid, but no one went to jail.^7^ Purdue Pharmaceuticals used fabricated data to get its long-acting oxycodone (OxyContin®) approved, and thousands of people became addicted and died; fines were paid, but no one served jail time.^8^ Rofecoxib (Vioxx®) was approved because Wyeth withheld data from the FDA showing that its drug quadrupled the risk of myocardial infarction; a fine was paid, but no one served jail time.^9^

Can we anticipate any major change? It is unlikely as long as the pharmaceutical industry controls the purse strings – not only of the FDA but of the government. There are more than two pharmaceutical lobbyists for every representative in Washington, D.C.^10^ The recent price rise in lifesaving drugs like epinephrine and naloxone show how powerless the government is in preventing patient harm.

The article in this month’s *West*JEM by Zuabi et al. is based on a talk that I developed about 10 years ago but was seldom asked to give. When I gave it at the American Academy of Emergency Medicine Scientific Assembly in 2016, *West*JEM Editor-in-Chief Mark Langdorf was in the audience and became intrigued. I was unable to dedicate the time to write the article but sent all of my references and resources, and this article was what came out of it. They did a great job of synthesizing my talk into a fascinating article, far better than I could have. I congratulate the authors.
